# FullyPrinted Flexible Plasmonic Metafilms with Directional Color Dynamics

**DOI:** 10.1002/advs.202002419

**Published:** 2020-11-25

**Authors:** Jialong Peng, Hyeon‐Ho Jeong, Michael Smith, Rohit Chikkaraddy, Qianqi Lin, Hsin‐Ling Liang, Michael F. L. De Volder, Silvia Vignolini, Sohini Kar‐Narayan, Jeremy J. Baumberg

**Affiliations:** ^1^ NanoPhotonics Centre, Cavendish Laboratory University of Cambridge Cambridge CB3 0HE UK; ^2^ Department of Materials Science & Metallurgy University of Cambridge Cambridge CB3 0FS UK; ^3^ Institute for Manufacturing Department of Engineering University of Cambridge Cambridge CB3 0FS UK; ^4^ Department of Chemistry University of Cambridge Cambridge CB2 1EW UK; ^5^ Present address: School of Electrical Engineering and Computer Science Gwangju Institute of Science and Technology Gwangju 61005 Republic of Korea

**Keywords:** active dichroism, flexible plasmonics, hybrid nanophotonics, nano‐printing, printed metamaterials

## Abstract

Plasmonic metafilms have been widely utilized to generate vivid colors, but making them both active and flexible simultaneously remains a great challenge. Here flexible active plasmonic metafilms constructed by printing electrochromic nanoparticles onto ultrathin metal films (<15 nm) are presented, offering low‐power electricallydriven color switching. In conjunction with commercially available printing techniques, such flexible devices can be patterned using lithography‐free approaches, opening up potential for fullyprinted electrochromic devices. Directional optical effects and dynamics show perceived upward and downward colorations can differ, arising from the dissimilar plasmonic mode excitation between nanoparticles and ultrathin metal films.

## Graphical abstract

Commercially available printing techniques offer fullyprinted flexible plasmonic metafilms, which show electrically‐tunable omni‐ or bi‐directional color dynamics engineered with structural geometries. Such activelycontrolled directional color dynamics in flexible plasmonics significantly unlocks the full potential of plasmonics for flexible/wearable devices.

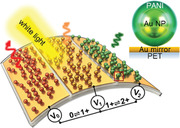

## 1 Introduction

Active plasmonic coloration describes the tunable structural pigmentation generated by metallic nanostructures and has emerged as a critical application space for plasmonics.^[^ ^1^, ^2^ ^]^ Recently, an advanced concept in plasmonic nanopixels, based on electrochromic nanoparticle‐on‐mirror constructs^[^ ^3^, ^4^ ^]^ (*e*NPoM), has demonstrated strong vivid color dynamics across the visible spectrum with fast switching speeds (<100 ms) and low energy consumption (<0.3 mW cm^−2^). A significant potential advantage is that their fabrication can be scaled from the single nanoparticle level to meter‐scale metasurfaces via lithography‐free methods. As a result, these hold promise for applications from “always‐on” electronic shelf labels to low‐energy‐consumption color‐changing films, but key challenges remain to unlock this vision.

One highlydesired feature for device configuration is large‐area flexibility, spanning from wearable devices^[^ ^5^, ^6^, ^7^ ^]^ to architectural films. Flexible static plasmonics can be formed via plasmonic nanostructures on plastic films,^[^ ^8^, ^9^ ^]^ but it has been challenging to make these colors controllable electrically. One popular approach is to position plasmonic elements onto deformable flexible materials, typically elastomeric polymers.^[^ ^10^, ^11^, ^12^, ^13^, ^14^ ^]^ Mechanical deformation of these stretchable/bendable substrates modifies the inter‐element spacing, providing active optical tuning. However this limits their adoption since tuneability is limited and creating the forces required is nontrivial, particularly electrically. By contrast, combining plasmonic systems such as gratings^[^ ^15^ ^]^ or thin‐film cavities^[^ ^16^ ^]^ with stimulus‐responsive materials^[^ ^1^ ^]^ such as electrochromic^[^ ^2^, ^15^, ^16^, ^17^, ^18^ ^]^ or phase change materials,^[^ ^19^, ^20^, ^21^, ^22^ ^]^ promises electrically‐tunable color switching. However these devices are typically rigid and suffer from limited optical switching (solely “on‐off” function),^[^ ^15^, ^16^, ^17^, ^18^ ^]^ long response times (multi‐second)^[^ ^22^ ^]^ and/or poor long‐term reproducibility (<1 month).^[^ ^23^ ^]^

Large‐area nanopatterning is another challenge confronting the fabrication of these flexible systems. Heavy reliance on complex lithographic processes is needed, especially in designs requiring nanoscale precision at the wafer scale.^[^ ^24^ ^]^ Rastering techniques including electron‐ and focused‐ion beam lithographies^[^ ^15^, ^25^ ^]^ are the preferred choice for patterning nanostructures with high precision, but they are generally expensive, time‐consuming, and less compatible with flexible plastics. In contrast, imprinting and transfer approaches^[^ ^18^, ^26^, ^27^, ^28^, ^29^ ^]^ can pattern target structures on large‐area plastic repeatedly. However, their required pre‐determined master templates still rely on rastering techniques, which are expensive and vulnerable to defects (or contamination).^[^ ^30^ ^]^ A plausible solution to tackle such limitations is using nozzle‐based printing techniques including 3D, inkjet, and aerosol‐jet printing. Such methods have emerged as key for flexible and wearable electronics.^[^ ^31^ ^]^ In particular, with inks containing functional nanoparticles, very thin and uniform nanostructured electronic films can be directly deposited on large‐area or flexible substrates, and even 3D objects.^[^ ^32^ ^]^ While there are a few examples of printed static plasmonics, such as surface‐enhanced Raman scattering substrates,^[^ ^33^ ^]^ or thermo‐plasmonic interfaces,^[^ ^34^, ^35^ ^]^ there have been no demonstrations for flexible electrochromic plasmonic systems.

Here we demonstrate all‐printed flexible active plasmonic metafilms, which address the two challenges above together, namely flexibility and large‐area nanopatterning. Large‐scale flexible active plasmonic metafilms are fabricated by depositing *e*NPoM structures onto ultrathin metallized plastic films via printing and coating. The printed colors are not only uniform across tens of centimeters (scale limit of our commercial printer), but also sustain their performance while bending the supporting plastic film underneath. Depending on the interaction with the direction of propagating light, such metafilms give rise to directional plasmonic coloring effects and dynamics induced by the thin‐mirror configuration. This suggests that such tunable directional color dynamics developed in all‐printed plasmonic devices can unlock the full potential of flexible plasmonics for industrial applications.

## 2 Results and Discussions

### 2.1 Optical Dynamics of Electrochromic Nanoparticle‐on‐Mirror Metafilms

**Figure 1:**
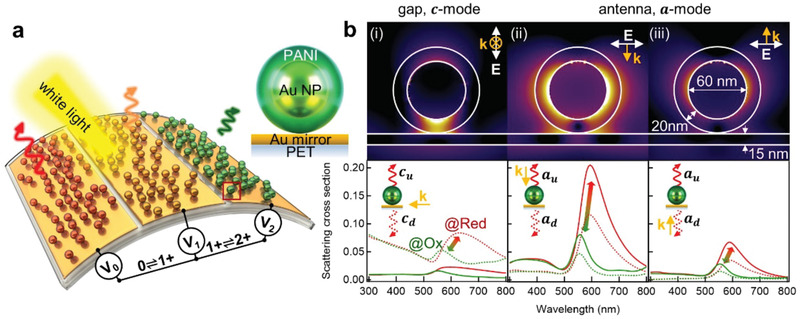
Active plasmonic metafilms with ultrathin mirror. a) Schematic of flexible active plasmonic metafilms, with color changes set by the redox state of the polyaniline (PANI) (*V*_0_: fully reduced, *V*_1_: half oxidised, *V*_2_: fully oxidised). Right panel shows individual *e*NPoM. b) Optical near‐field enhancement of the *e*NPoM for reduced state of PANI, with different polarized light illumination (i, gap mode, ii,iii, antenna modes, upper panel), and their corresponding upward (solid lines) and downward scattering (dashed lines, bottom panel), via numerical simulation (see Section 4).

The active plasmonic metafilms are formed by transferring electrochromic nanoparticles consisting of ≈ 60 nm gold nanoparticles (Au NP) encapsulated in a continuous conducting polymer shell (≈ 20 nm), here polyaniline (PANI), onto a flexible gold‐coated polyethylene terephthalate (Au‐PET) film (**Figure** **1**a). These form a nanoscale multilayer of Au NP‐PANI‐Au‐PET, termed an *e*NPoM, where the PANI layer acts as the gap between the Au NP and Au‐PET film. Light is strongly confined in this gap, amplifying the plasmonic coloration. Moreover, this can be tuned in a controlled manner by switching the redox state of the PANI shell electrically, through the adjacent Au‐PET substrate. When the redox state of the PANI layer changes from reduced to oxidized states (Figure 1b), numerical simulations of single *e*NPoMs show the scattering spectra shift from red to green (≈ 70 nm wavelength shift) due to the change in the effective refractive index of the PANI shell, with corresponding 60% intensity changes (Figure S1, Supporting Information, for experimental scattering spectra of single *e*NPoM).^[^ ^36^ ^]^

One approach to make such films flexible is through an ultrathin metal mirror configuration, using very thin and continuous metal films on the PET. Initial theoretical investigations explore how metal layers thin enough to be bendable can still act as mirrors to plasmonically confine light inside the gap. When illuminated from i) side‐on, ii) above, or iii) the underside of the *e*NPoM, different plasmonic modes are excited which present similar spectral dynamics in all cases (red to green according to the change in the redox state of the PANI shell upon cycling *V*_0_↔*V*_2_, Figure 1b). For *e*NPoMs with Au mirror thickness *h*  = 15 nm, the case (i) where incident light is polarized perpendicular to and propagating along the Au‐PET film shows the optical field is strongly confined in the gap between the Au NP and the Au‐PET film (known as a “hot spot”).^[^ ^37^, ^38^ ^]^ This results in a strong coupled‐gap resonance (*c*‐mode ≈ 631 nm peak wavelength at *V*_0_) which redshifts by ≈ 50 nm for *V*_0_ → *V*_2_ in both upward (*c*_u_) and downward (*c*_d_) scattering with maximum *c*_d_ > 200% higher than *c*_u_ (Figure 1b i). This is because the *c*‐mode is almost independent of mirror thickness (even when below the skin depth of gold, ≈ 40 nm),^[^ ^39^ ^]^ but the light penetrates through the thin mirror and scatters downwards, rather than entirely up‐scattering as for thick Au mirrors (>45 nm).^[^ ^4^, ^39^, ^40^ ^]^ On the other hand when illuminated from above (Figure 1b ii) or below (Figure 1b iii), its transverse antenna *a*‐mode is excited, appearing weaker from below since the incident light suffers absorption in the mirror. No matter which illumination direction,^[^ ^39^ ^]^ the upward scattering intensity *a*_u_ is almost double the downward direction *a*_d_, due to the radiation pattern of this mode. In all three cases, no matter which modes are excited, identical coloration and dynamics in scattering are predicted (Figure S2, Supporting Information). This shows that the valuable properties of *e*NPoMs are preserved in the thin‐metal film version required for flexible devices, and that the scattering is independent of direction, as required for ambient‐light reflective devices.

### 2.2 Fabrication of Flexible Electrochromic Nanoparticle‐on‐Mirror Metafilms

**Figure 2:**
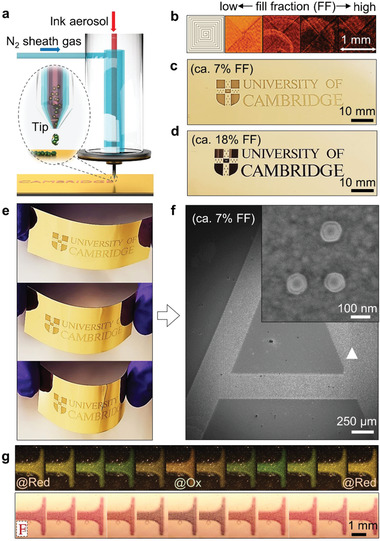
Printed flexible *e*NPoM metafilms. a) Schematic of aerosol‐jet printing. b) Bright field (BF) images with different printing fill fractions (FF), and example printings with c) FF = 7% and d) FF = 18%. e) Photos of a printed flexible plasmonic film under bend testing. f) Scanning electron microscopic (SEM) image of printed character “A.” Inset shows core‐shell structure of PANI‐coated Au nanoparticles. g) DF and BF optical images of part of printed character “F” versus voltage applied from −0.15↔0.65 V and back (FF = 7% sample).

We fabricate flexible *e*NPoM metafilms by using an aerosol‐jet printing technique (**Figure** **2**a). This enables large‐area fine‐feature nanoparticle patterning on both rigid and flexible substrates using a broad choice of functional inks.^[^ ^32^, ^41^ ^]^ The printing ink is prepared by concentrating as‐prepared PANI‐coated Au NP solution to ≈ 5 × 10^11^ NP mL^−1^ (see Section 4). It is worth noting that while this ink is concentrated, its nanoparticle density is still significantly lower than that of conventional nanoparticle inks used with aerosol‐jet printing (≈1 × 10^14^ NP mL^−1^). Here, the objective is instead to produce a sparse monolayer distribution of nanoparticles, rather than the dense conducting tracks typical of printed electronics. We emphasize that the precise location of individual nanoparticles cannot be controlled using such printing methods, however, the patterning of metafilms only requires a monolayer of “randomly”distributed nanoparticles within each pixel with the nm‐gap thickness control provided by the nanoparticle coating. A 1 mL reservoir of the PANI‐coated Au NP ink is atomized into aerosol droplets via ultrasonic atomization. The ink‐containing aerosol is carried by nitrogen gas at a flow rate of ≈ 30 standard cubic centimeters per minute (SCCM) and jetted through the printer nozzle (diameter 150 µm) assisted by a nitrogen sheath flow (at ≈ 40 SCCM) to focus it onto the substrate. The printed line width is ≈ 12.5 µm under these conditions and can be tuned by adjusting the gas flow rates and nozzle tip size (Figure S3, Supporting Information).

A sparsely distributed fractional monolayer of *e*NPoMs is formed within the printing line. Sparse aggregates form because of capillary forces during drying of the ink droplets or between overcoats via multiple printing, but this does not influence the color appearance as the optical coupling between neighboring nanoparticles is minimized by their >40 nm thick polymer separation.^[^ ^3^, ^4^ ^]^ The Au‐PET substrate is fixed on a translation stage moving at speeds of 1 mm s^−1^, which determines the printing speed. A square loop test pattern (1 mm^2^) is used to explore the optimal printing conditions in order to get uniform color appearance across the patterned area with minimal printing time (here 78 s, Figure 2b). The *e*NPoM fill fraction in the printed area (the areal number density of nanoparticles) can be tuned by simply overprinting the layer multiple times (Figure S3, Supporting Information). Alternatively, controlling the nanoparticle concentration in the ink can enhance the printing speed but can lead to creation of more *e*NPoM aggregates in the film.^[^ ^32^ ^]^

A simple university logo is printed on 15 nm thick Au layer PET films with 60 nm diameter Au NPs coated with 20 nm thick PANI, for proof‐of‐concept flexible devices (Figure 2c,d, Figure S4, Supporting Information). The nanogap between the Au NP and the thin‐mirror is precisely controlled by the thickness of the PANI shell via the wet chemistry growth (inset of Figure 2f, Figure S5, Supporting Information).^[^ ^3^ ^]^ Since these nanogaps critically determine the color of the film (spectral shifts of 5 nm per 1 nm increase in gap size) and are extremely monodispersed, their coloration is not only vivid under ambient light (for only 7% fill fraction of NPs), but also remains uniform over multi‐centimeter length scales while bending the film, never previously possible with any plasmonic system (Figure 2e).

When the redox state of PANI is changed electrochemically (*V*_0_↔*V*_2_) using the bottom ultrathin metal, vivid uniform color dynamics can be observed under both dark field (DF) and bright field (BF) illumination (Figure 2g; for other printed examples with different sizes of Au NPs see Figure S6, Supporting Information). With further engineering of the rheological properties of the ink, this printing method can be adapted for commercial large‐scale industrial printing rigs, or consumer household printers (in progress), leading to widespread customized flexible and wearable plasmonic electrochromic devices.

### 2.3 Omnidirectional Coloration in Electrochromic Nanoparticle‐on‐Mirror Metafilms

Color differences between DF and BF scattering (apparent in Figure 2g) are due to the contributions of light absorption and reflection from the *e*NPoM. We thus now systematically investigate the directional coloring effects in plasmonic metaflims while reducing the Au mirror thickness. Centimeter‐scale *e*NPoM metafilms with different thickness of Au mirror (*h* = 5, 10, 15 nm) are first fabricated via meniscus‐guided nanoparticle assembly (see Section 4 and Figure S7, Supporting Information), where *e*NPoMs are also randomly distributed (Figure S8, Supporting Information). When large‐area patterning is secondary, this meniscus‐guided nanoparticle assembly is used since only ≈ 20 µL of the ink is required for small areas (≈ 1 cm^2^) of uniformlycolored *e*NPoM metafilms.^[^ ^42^, ^43^ ^]^ The fill fractions of the metafilms are tuned by controlling the coating parameters including particle concentration in suspension (Figure S9, Supporting Information).^[^ ^3^, ^32^, ^44^ ^]^

**Figure 3:**
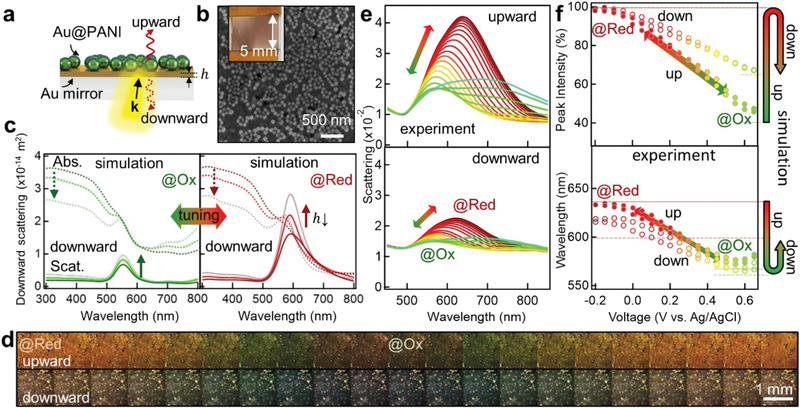
Omnidirectional coloration in scattering. a) Schematic of *e*NPoM metafilm and b) SEM image of film coated using meniscus‐guided assembly. Inset shows corresponding optical image. c) Simulated downward scattering (solid line) and absorption (dashed line) spectra for oxidation (green) and reduction (red) states of PANI with different Au thickness (15 nm, 10 nm, 5 nm from dark to faint lines). d) Experimental DF upward‐ (top) and downward‐scattering images (bottom) of the *e*NPoM metafilm with 5 nm Au thickness, and e) corresponding scattering spectra and f) coupled mode peak intensity and wavelength change versus applied voltage −0.2↔0.65V. The transparent lines indicate simulated results for oxidation (green) and reduction (red) states of PANI (solid lines correspond to upward scattering, and dashed lines are downward scattering).

The scattering of the *e*NPoMs display distinct chromatic directionality, changing with incident direction and Au mirror thickness (**Figure** **3**). We first examine back‐illumination where the *e*NPoM color appearance hinges on the wavelength and intensity of the light coming through the mirror (Figure 3a). For comparison, the calculated scattering and absorption of *e*NPoMs with different Au mirror thickness at different redox states of the PANI shell is evaluated (Figure 3c, Figure S10, Supporting Information). When PANI is oxidized (*V*_2_), the absorption overwhelms the scattering (over two‐fold), thus removing the color appearance for all mirror thicknesses. In the reduced state of PANI (*V*_0_), the scattering increases and overcomes the absorption for Au mirror thickness below 10 nm. Omnidirectional scattered colors and tuning are thus predicted in the *e*NPoM metafilm with thin Au mirrors (here 5 nm), with the capability for dimming (or blocking) the downward scattering by controlling the mirror thickness.

Experimentally, reversible color dynamics and optical switching are indeed observed in the *e*NPoM metafilm with 5 nm Au thickness (DF images, Figure 3d). The upward scattering peak shifts from 633 nm to 572 nm when *V*_0_ → *V*_2_, losing ≈ 100% intensity. The downward scattering gives similar trends but with reduced tuning range, shifting from 616nm to 565 nm with ≈ 46% intensity loss (Figure 3e,f). The film is thus omnidirectionally colored in the oxidized state. This shows the capability to tune from omnidirectional coloration (for thin mirrors) to single‐sided coloration (for thick mirrors), solely using the Au mirror thickness (for other Au mirror thickness, see Figure S11, Supporting Information).

### 2.4 Bidirectional Coloration in Electrochromic Nanoparticle‐on‐Mirror Metafilms

**Figure 4:**
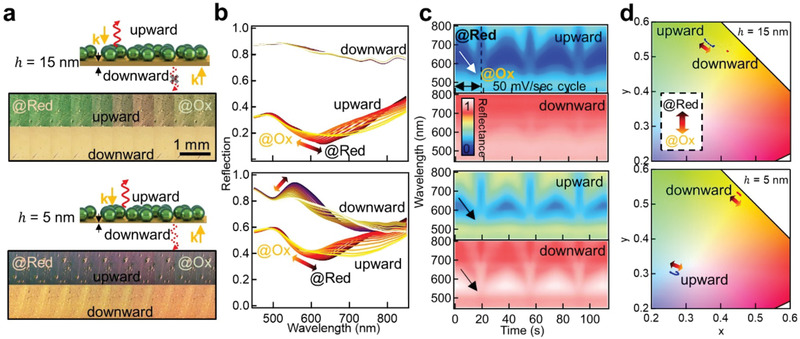
Bidirectional coloration in reflection. a) Schematic of the *e*NPoM metafilms with thick (15 nm, top panel) and thin Au layers (5 nm, bottom panel) and their corresponding BF optical images in up‐ and downward reflection. b) Normalized reflection spectra versus voltage change. c) Time scans of normalized reflection spectra for 3 cycles of voltage −0.2 ↔ 0.6 V at scan rate 50 mV s^−1^, and d) corresponding color gamut dynamics (CIE 1964).

The omnidirectional plasmonic coloring in scattering from the thin‐mirror configuration can be transformed into bidirectional color dynamics by incorporating reflection effects (**Figure** **4**). Samples are observed in bright‐field to quantify how additional surface light reflection from the surrounding mirror changes the color appearance of the *e*NPoM metafilms. Metafilms with both *h* = 15 nm (top panel Figure 4a) and *h* = 5 nm thick Au mirrors (bottom panel) show vivid uniform color modulation in the upward reflection from ≈ 580 nm to ≈ 620 nm when the external voltage is switched between *V*_0_↔*V*_2_ (Figure 4b for reflection spectra). By contrast, the downward reflection of the metafilm depends on mirror thickness: 15 nm thick Au acts as a near‐complete mirror, reflecting all light and appearing golden with negligible electrochromic tuning. For the 5 nm thick Au, a stronger color dynamic is observed from 545 to 557 nm that differs from the up‐reflected tuning (Figure 4c,d show color gamut plots). This dynamical tuning is highly reproducible over >100 cycles of *V*_0_↔*V*_2_. A fully reversible bidirectional color dynamic tuning is thus achieved in a plasmonic system. While commercial applications of dichroism such as dichroic glass are generally based on thin‐film interference^[^ ^45^ ^]^ whose structures are static and rigid,^[^ ^46^, ^47^, ^48^, ^49^ ^]^ these *e*NPoM metafilms are flexible and electrically‐tunable. They thus compare favorably with the original 4th century demonstration seen in the “Lycurgus Cup.” This bidirectional tunable color dichroism could thus be developed for flexible active dichroic devices.

## 3 Conclusion

We demonstrate large‐area flexible active plasmonic metafilms, which show electrically tunable vivid color dynamics without relying on mechanical deformation or grating‐like structures. In conjunction with commercially available printing, metafilms comprising of a monolayer of electrochromic nanoparticles (<100 nm) can be laiddown uniformly and patterned precisely within each pixel, offering possibilities to make all‐printed wearable plasmonic devices including devices such as electronic shelf labels/indicators, electrochromic textiles integrated with clothes, or biochemical sensors for detecting the protonation level in skin sweat.^[^ ^50^, ^51^ ^]^ The whole process is lithography‐free and thus readily extends to industrial large scale processing tools.^[^ ^52^ ^]^ In addition, using directional optical effects that can be controlled with this ultrathin mirror configuration, such flexible active plasmonic metafilms can generate electrically controllable omni‐ or bi‐directional color dynamics, which could be used for “magic‐mirror” applications, color‐tunable glass, and architectural decoration. Although the presented colors are currently limited, they can be further extended by engineering the gap materials (for instance with alternative or blended conducting polymers or other responsive organic/inorganic/hybrid materials) or the plasmonic materials (utilizing other metals such as Ag, Al, or dielectric scatters).

## 4 Experimental Section

##### Electrochromic Nanoparticle‐on‐Mirror

PANI was coated on the surface of Au NPs using surfactant‐assisted chemical oxidative polymerization.^[^ ^3^, ^53^ ^]^ First, 1.6 mL of the citrate‐stabilized Au NPs (from BBI) was concentrated and the supernatant was discarded. Then 0.12 mL of 40 mm sodium dodecyl sulfate (SDS) and 0.6 mL of 2 mm aniline were added. After vigorous mixing, the polymerization took place upon adding 0.6 mL of 2 mm ammonium persulfate in 10 mm hydrochloric acid into the solution. Incubated at room temperature overnight, the solution was washed with 4 mm SDS solution. The *e*NPoM devices were fabricated by placing the nanoparticle solution onto a metal mirror, via drop‐casting/coating/printing.

##### Metallic Thin Mirror Film

The Au‐PET films were commercial products from Eastman Flexvue (Au2, Au5, Au10). A planar gold layer on silicon substrates was prepared by evaporating 100 nm thick Au layer with 10 nm chromium adhesion layer onto a silicon wafer at a growth rate of 0.1 nm s^−1^ using an e‐beam evaporator (Lesker LEV).^[^ ^54^ ^]^ All coating substrates were cleaned with oxygen plasma before coating/printing.

##### Electrochemistry

A three‐electrode system was used for the electrochemical cell, connected with a potentiostat (Ivium Technologies): The Au substrate layer was used as a working electrode, with a Ag/AgCl reference electrode (Green Leaf Scientific) and a platinum mesh counter electrode (Alfa Aeser). All three electrodes were inserted into a fluid chamber, created by adhering two clean glass coverslips with a stack of double‐sided tape and filling with electrolyte solution (0.5 m NaCl in 10 mm HCl). The coated/printed region of the sample was sandwiched between the coverslips and immersed into the electrolyte. For flexible devices, indium tin oxide coated PET film (60 Ω sq^−1^, Sigma‐Aldrich) was used instead of using glass coverslips, which also functions as the counter electrode (Figure S4, Supporting Information).

##### Aerosol Jet Printing

Unless otherwise specified the devices were produced by this printing method. The ink for aerosol jet printing was prepared by concentrating 48 mL of 60 nm PANI‐coated Au NPs to 1 mL, and decanting the supernatant. The ink was then placed in a glass vial based ultrasonic atomizer of the aerosol jet printer (AJ200, Optomec). This ink aerosol was carried by nitrogen gas, and fed toward the printing nozzle tip, where a second “sheath” nitrogen gas flow was introduced to focus the aerosol jet. All prints carried out for this study used a tip with a 150 µm diameter opening, although other tip sizes are available. The atomizer and sheath flow rates were adjusted independently to find optimal printing conditions and to control the width of the printed line. When using a 150 µm diameter tip, typical flow rates were around 20–50 SCCM and 30–90 SCCM for the atomizer and sheath flows, respectively. The printing substrate was fixed on a translational stage which moved beneath the print head. A print speed of 1 mm s^−1^ was used for all prints in this study. Repeated multiple printing was used to achieve higher fill fractions for patterned structures. No thermal post‐treatment of the samples was carried out after printing.

##### Meniscus‐Guided Nanoparticle Assembly

The coating of the *e*NPoM metafilm was carried out using a guided convective coating set‐up (Figure S7, Supporting Information).^[^ ^42^, ^43^ ^]^ Substrates were adhered onto a translation stage (ProScanII). The solution (around 40 µL) of the concentrated PANI‐coated Au NPs was injected between the substrate and a fixed fluoro‐silanized glass, with ≈ 300 µm fixed spacing. The coating was achieved by dragging the formed liquid meniscus on one edge across the substrates with translation of the stage (at 1 µm s^−1^).

##### Scanning Electron Microscopic Analysis

SEM images of the samples were obtained by a LEO 1530VP (Zeiss, accelerating voltage of 10 kV).

##### Optical Imaging and Spectroscopy

Optical BF/DF images and spectra of samples were obtained using a CCD camera (Infinity 2) and spectrometer (Ocean Optics QE65000) with 100 × objectives (Olympus MPlanFLN) in a customized microscope (Olympus BX51). A halogen lamp (Philip 7023) was used as the white light source. Reflection and transmission measurements were conducted in BF configuration, and scattering measurements were conducted in DF configuration.

##### Numerical Simulation

The simulations of the electromagnetic response of the *e*NPoM use finite‐difference time‐domain calculation software (Lumerical Solutions). The Au nanosphere with a spherical PANI shell was placed onto a gold rectangular thin layer with specific thickness (refractive index from literature^[^ ^36^, ^55^ ^]^). The illumination source was a plane wave injected 100 nm away from the nanoparticle which was centered inside a 500 nm × 500 nm × 500 nm simulation box. The plane wave was oriented in the direction toward the nanoparticle core, for both side‐on and top/bottom illumination cases. The polarization angle was set as depicted in the corresponding schematics. The refractive index of the PET film is *n* = 1.65, and the whole surrounding environment is set as water (*n* = 1.33), to simplify the simulations.^[^ ^56^ ^]^

##### Statistical Analysis

The sample size was obtained from 25 PANI‐coated Au NPs in the SEM analysis, with an average PANI thickness of 19.4 ± 3 nm (data presented as mean ± standard deviation, see Figure S5, Supporting Information). Statistical analysis was carried out using Igor Pro8 Software (WaveMetrics).

## Conflict of Interest

The authors declare no conflict of interest.

## Author Contributions

J.P., H.‐H.J., and J.J.B. conceived of and developed the experiments, and together with M.S. and S.K.‐N. conducted the printing process; R.C. and J.P. undertook the modelling; Q.L. developed the electrochemistry; H.‐H.J., H.‐L.L., M.D.V., and S.V. developed scaled‐up systems.

## Data and Materials Availability

All data used for the figures available online in the Cambridge data repository: https://doi.org/10.17863/CAM.57674.

## Supporting information

Supporting Information
