# Supplementary material for: FullyPrinted Flexible Plasmonic Metafilms with Directional Color Dynamics
Source: Adv Sci (Weinh). 2020 Nov 25;8(2):2002419. doi: 10.1002/advs.202002419 (PMC7816707; doi:10.1002/advs.202002419)
Supplement: Supplementary file 1 — Supporting Information [file ADVS-8-2002419-s001.pdf]

WILEY-VCH

© 2020 Wiley-VCH GmbH

ADVANCED SCIENCE
Open Access

Supporting Information

for *Adv. Sci.*, DOI: 10.1002/advs.202002419

Fully-Printed Flexible Plasmonic Metafilms with Directional Color Dynamics

Jialong Peng,[1] Hyeon-Ho Jeong,[1†] Michael Smith,[2] Rohit Chikkaraddy,[1] Qianqi Lin,[1] Hsin-Ling Liang,[1,3] Michael F.L. De Volder,[3] Silvia Vignolini,[4] Sohini Kar-Narayan,[2*] Jeremy J. Baumberg,[1*]*

WILEY-VCH

© 2020 Wiley-VCH GmbH

Supporting Information

Fully-Printed Flexible Plasmonic Metafilms with Directional Color Dynamics

Jialong Peng,[1] Hyeon-Ho Jeong,[1†] Michael Smith,[2] Rohit Chikkaraddy,[1] Qianqi Lin,[1] Hsin-Ling Liang,[1,3] Michael F.L. De Volder,[3] Silvia Vignolini,[4] Sohini Kar-Narayan,[2*] Jeremy J. Baumberg,[1*]*

[1] Cavendish Laboratory, University of Cambridge, Cambridge CB3 0HE, UK
[2] Department of Materials Science and Metallurgy, University of Cambridge, Cambridge CB3 0FS, UK
[3] Institute for Manufacturing, Department of Engineering, University of Cambridge, Cambridge CB3 0FS, UK
[4] Department of Chemistry, University of Cambridge, Cambridge CB2 1EW, UK

* Correspondence should be addressed to: jeong323@gist.ac.kr, sk568@cam.ac.uk, jjb12@cam.ac.uk

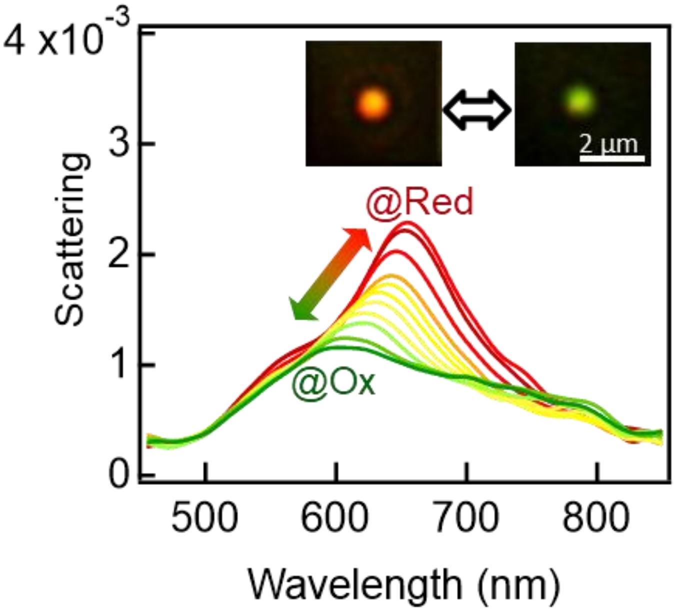

Figure S1: Experimental DF scattering spectra of a single *e*NPoM on 15 nm Au thickness Au-PET film *vs* applied voltage -0.2↔0.65V. Insets show the corresponding DF images at V_0 (left) and V_2 (right).

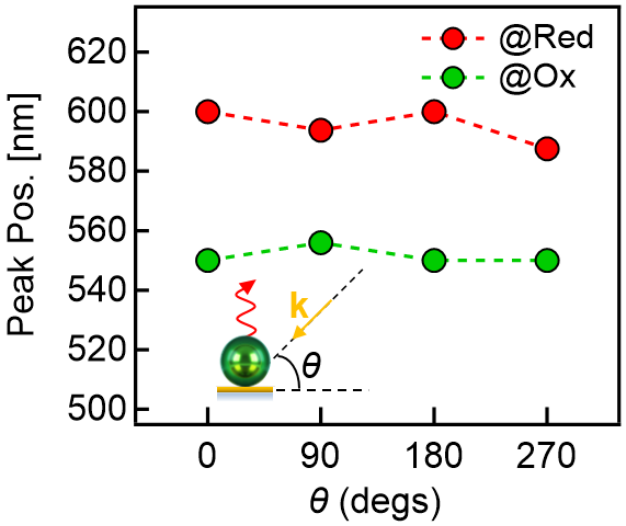

Figure S2: Simulated peak positions at reduced and oxidized states as a function of angle θ between the illumination source input direction **k** and the mirror plane.

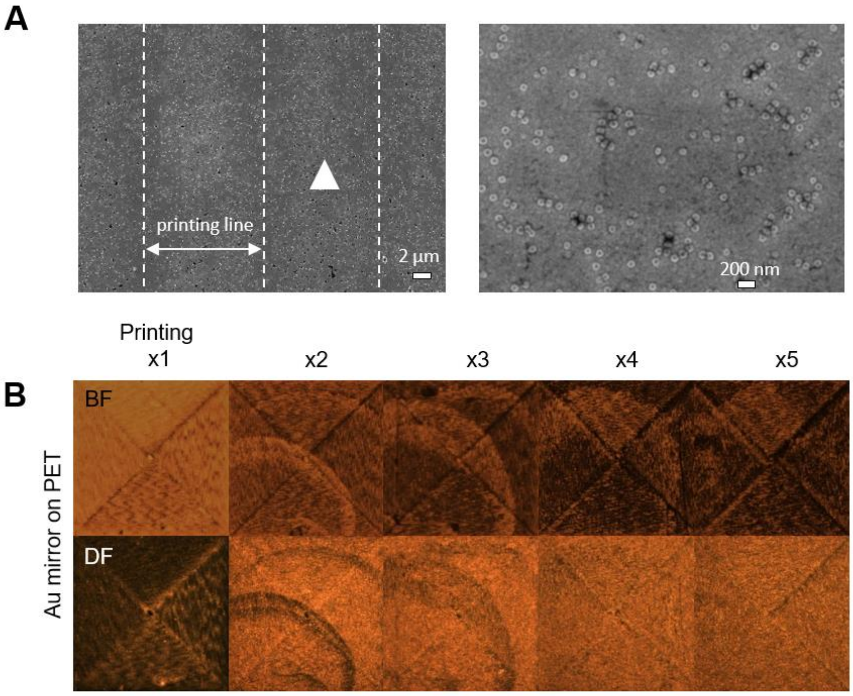

Figure S3: (A) SEM images of printed lines, right panel is zoomed-in image area indicated by the white triangle. (B) Bright field (BF, upper panel) and dark field (DF, lower panel) images of the printed *e*NPoM metafilms on Au-PET using aerosol jet printing. From left to right shows increasing overwriting series from 1-5.

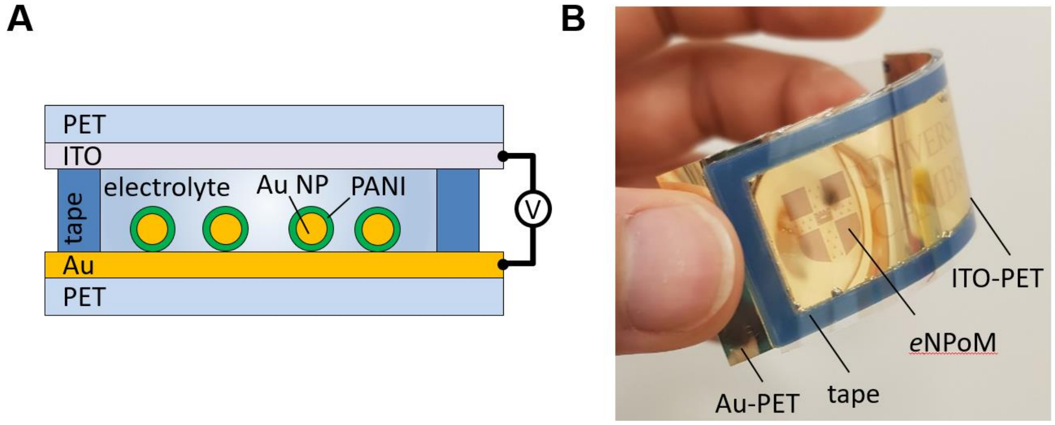

Figure S4: Large area flexible active plasmonic devices. (A) Schematic side view of the device configuration. (B) Image of demonstration device.

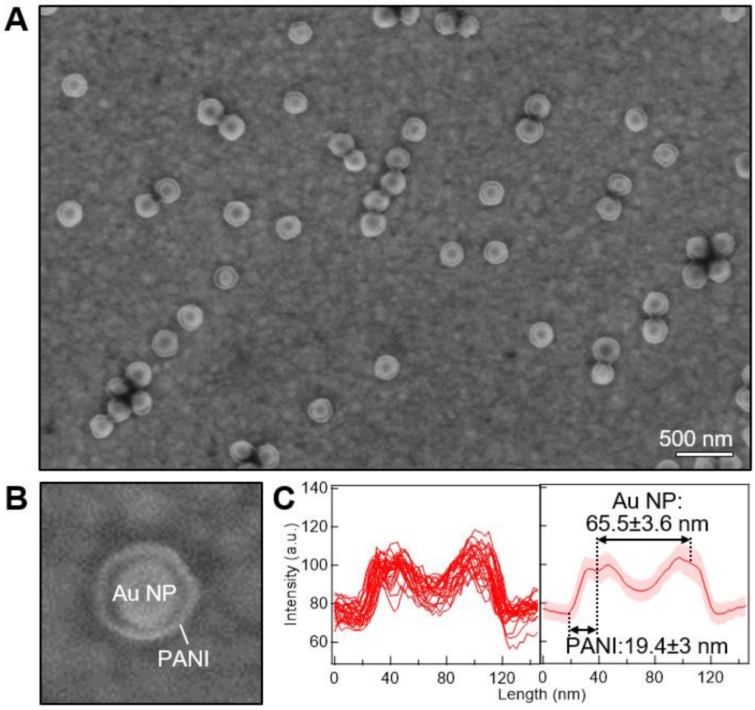

Figure S5: PANI coating on Au NPs. (A) SEM images of PANI-coated Au NPs on Au-PET and (B, C) close-up image of single Au NP (65 nm) with 19 nm thick PANI shell. Data presented as mean ± standard deviation, sample size = 25.

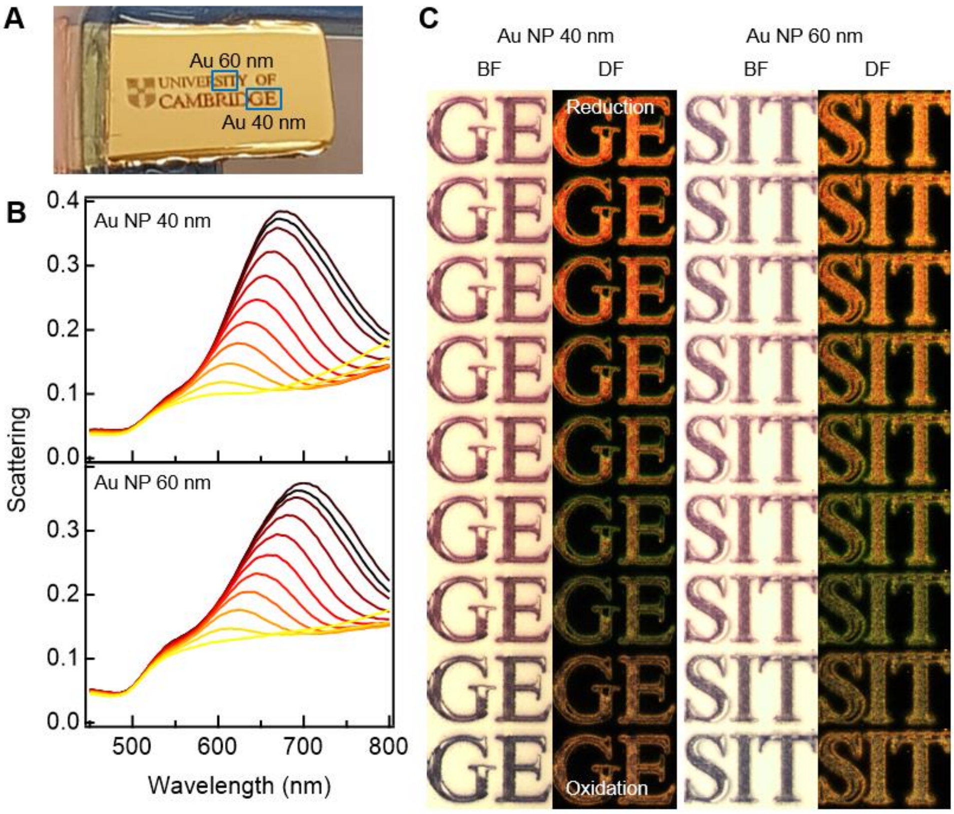

Figure S6: Printed patterns of *e*NPoMs on evaporated Au film. (A) Printed sample with different sizes of PANI-coated Au NPs and (B) their corresponding DF scattering spectra, and (C) BF and DF optical images when the voltage is ramped from -0.15 → 0.65 V.

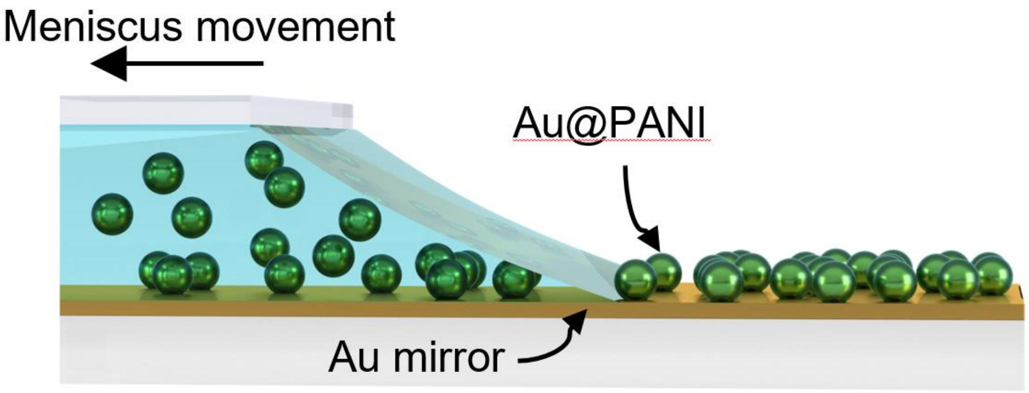

Figure S7: Schematic of meniscus-guided nanoparticle assembly (not to scale).

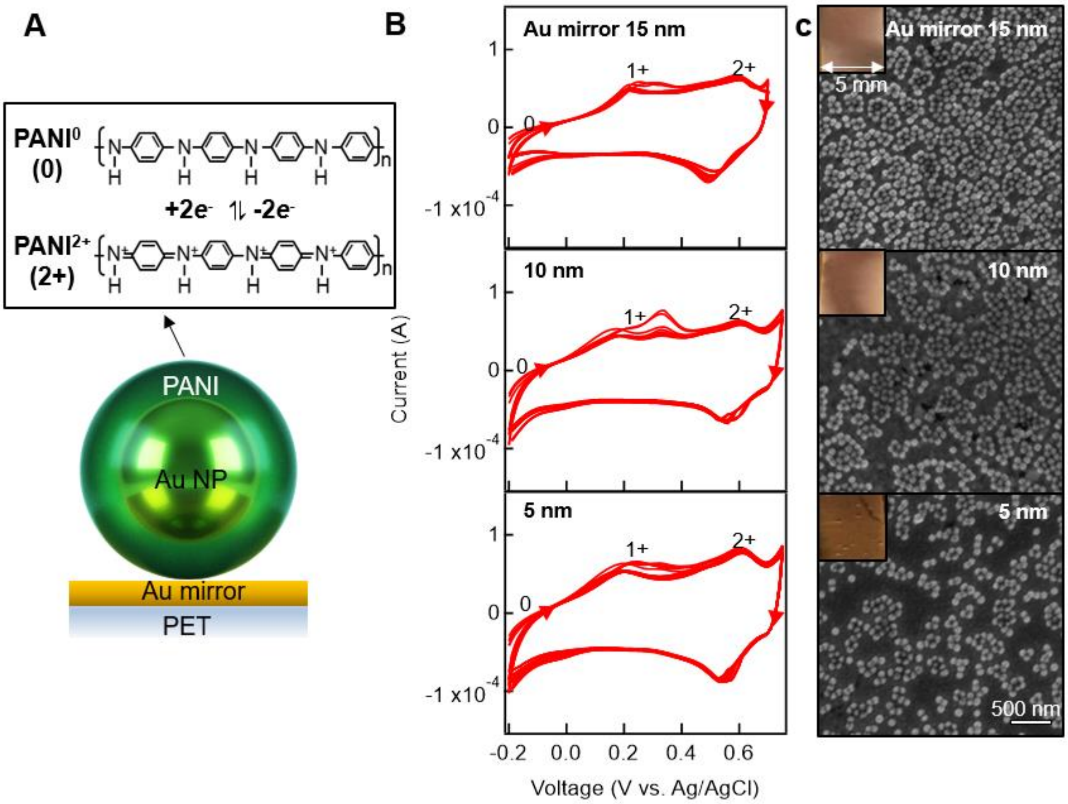

Figure S8: *e*NPoM electrochemistry. (A) PANI redox reactions. (B) CV curves of the *e*NPoM metafilms with different thickness Au layers (15, 10, 5 nm) on PET film, showing two sets of typical redox peaks (0: fully reduced, 1+: half oxidized, 2+: fully oxidized), and (C) corresponding SEM and optical images.

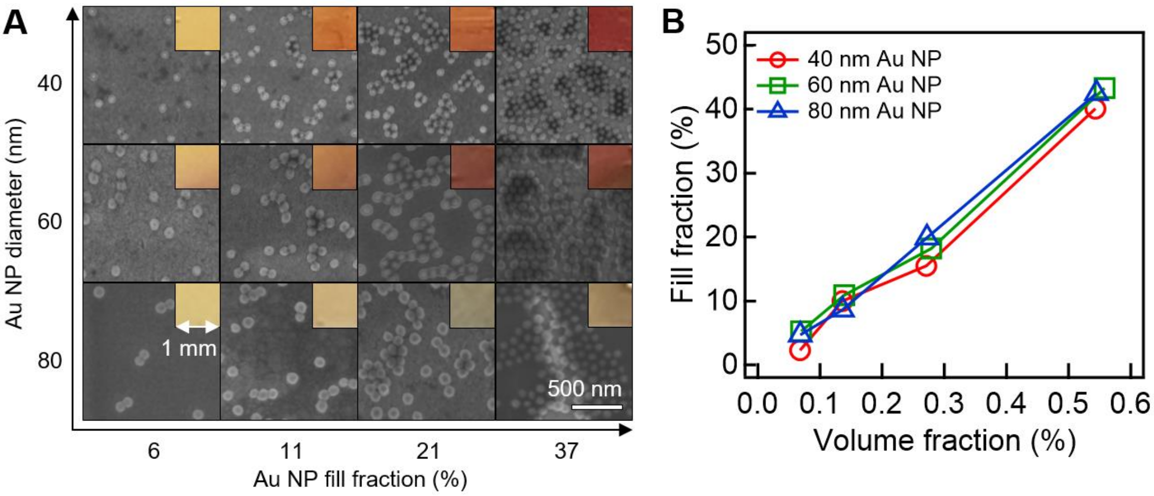

Figure S9: Meniscus-guided coating with various parameters (on evaporated Au film). (A) SEM images and optical images (insets) of coated *e*NPoM metafilms with different particle sizes (40, 60, 80nm Au NPs) and fill fractions. (B) Relationship between colloidal suspension volume fractions and final coating fill fractions for different particle sizes.

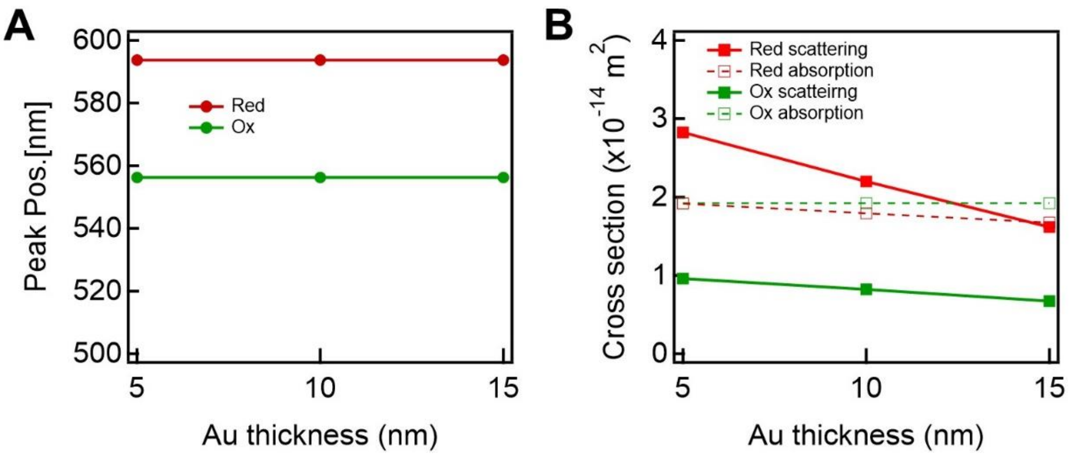

Figure S10: Simulated downward scattering, of (A) peak position for oxidation (green) and reduction (red) states of PANI with different Au thickness, and (B) corresponding scattering and absorption cross-sections. The color dynamics is similar for each Au thickness, but different in the intensity dynamics. The scattering overcomes the absorption for Au mirror thickness below 10 nm when PANI is in its reduced state. But when PANI is oxidized, the absorption overwhelms the scattering over two-fold, thus removing the color appearance for all mirror thicknesses.

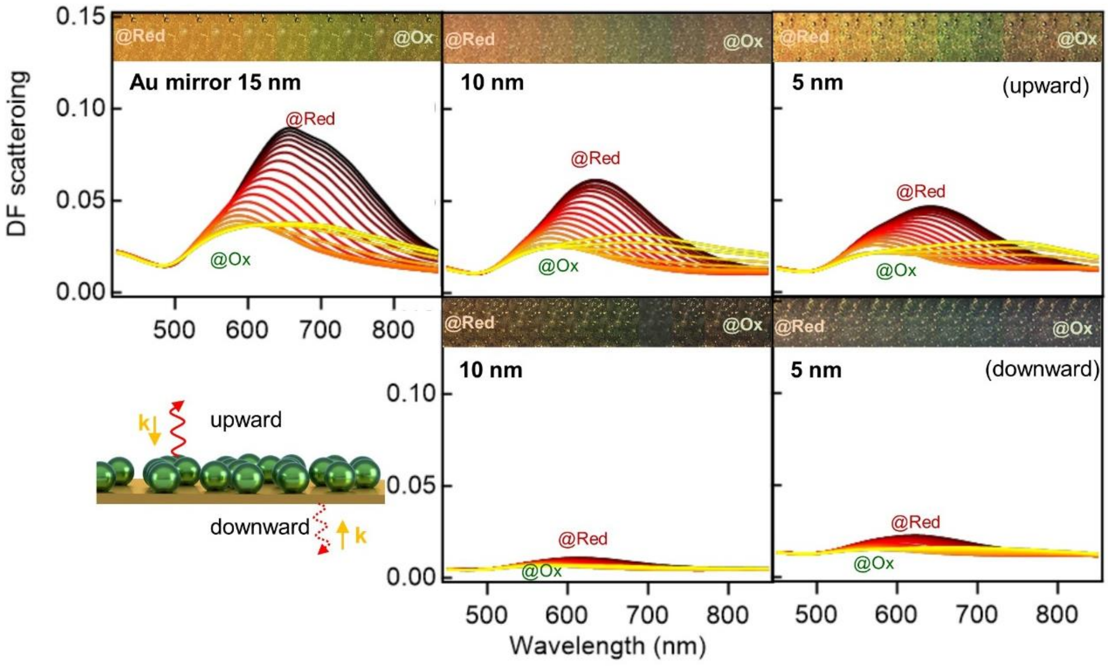

Figure S11: DF scattering dynamics and images (insets) of the *e*NPoM metafilms with different Au mirror thickness (left to right: 15, 10, 5 nm) on both sides (top panel: forward, bottom panel: backward).

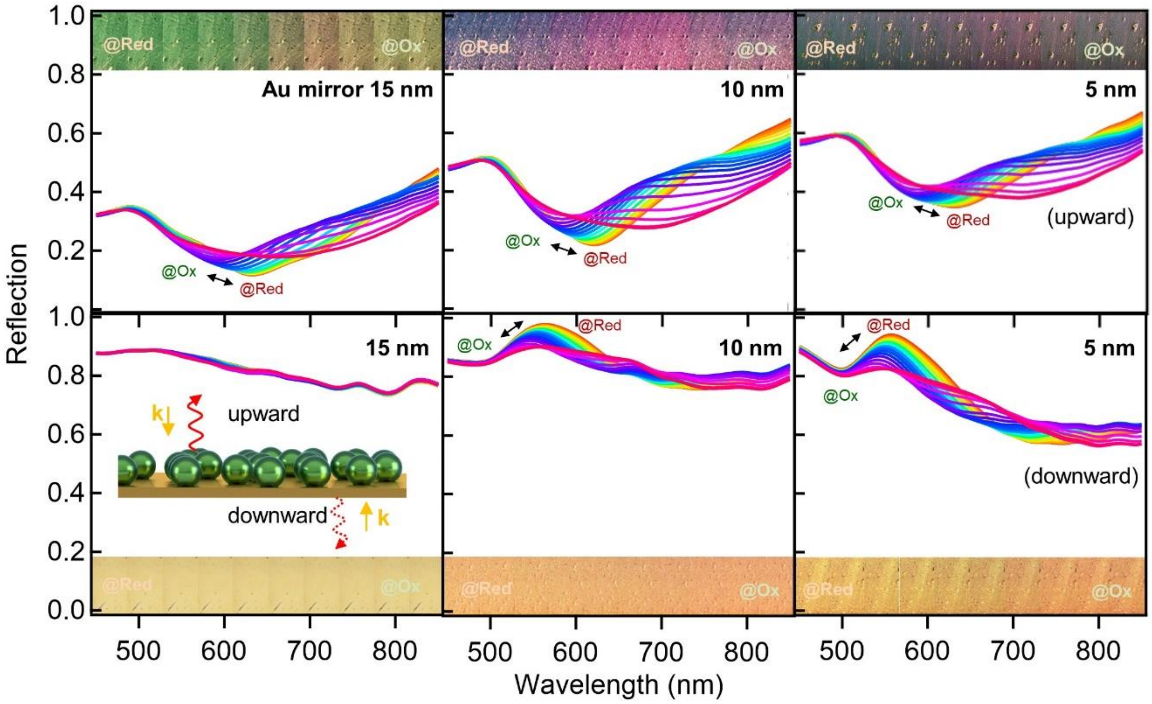

Figure S12: Color dynamics and images (insets) of the *e*NPoM metafilms in BF reflection on both sides (top: forward, bottom: backward) of different Au mirror thickness (left to right: 15, 10, 5 nm).
